# Physiological and Fitness Adaptations after Eight Weeks of High-Intensity Functional Training in Physically Inactive Adults

**DOI:** 10.3390/sports6040146

**Published:** 2018-11-13

**Authors:** Matthew F. Brisebois, Brandon R. Rigby, David L. Nichols

**Affiliations:** Department of Kinesiology, Texas Woman’s University, Denton, TX 76204, USA; MBrisebois@twu.edu (M.F.B.); DNichols@twu.edu (D.L.N.)

**Keywords:** CrossFit, HIFT, extreme conditioning programs, ECP, circuit training, multimodal training, MMT

## Abstract

The purpose of this study was to characterize high-intensity functional training (HIFT) in physically inactive adults. Four men and 10 women who were inexperienced with HIFT and not performing regular physical activity performed HIFT 3 days/week for 8 weeks. Health and fitness measures were assessed before and after the intervention. Resting heart rate (73 ± 12 vs. 68 ± 11 bpm) and resting diastolic blood pressure (71 ± 7 vs. 65 ± 6 mmHg) were reduced, while resting systolic blood pressure remained unchanged. Absolute VO_2max_ (2.53 ± 0.68 vs. 2.69 ± 0.66 L/min) and relative VO_2max_ (32.51 ± 8.84 vs. 34.31 ± 8.63 mL/kg/min) were improved. Lean body mass (48.20 ± 13.37 vs. 49.26 ± 13.81 kg) was increased, but fat mass was unchanged. Performance on the leg press (164.61 ± 54.35 vs. 201.62 ± 67.50 kg), bench press (39.12 ± 20.15 vs. 46.43 ± 21.18 kg), YMCA bench press (26 ± 13 vs. 37 ± 16 reps), one-minute sit-up (25 ± 9 vs. 32 ± 10 reps), and sit-and-reach (30.36 ± 11.36 vs. 32.14 ± 9.66 cm) were all increased. High-intensity functional training may be useful for improving health-related physical fitness parameters in physically inactive adults.

## 1. Introduction

The prevalence of health complications and chronic diseases associated with physical inactivity have continued to increase over the past few decades [1]. There is a strong relationship between inactivity and metabolic diseases, including obesity [2]. Indeed, the age-adjusted prevalence of obesity was 37.7% in 2014, an increase from 33.8% in 2008 [3,4]. Amidst increasing sedentary behaviors in modern society, the importance of engaging in regular physical activity must be emphasized. Regular physical activity remains one of the most powerful treatments in combatting obesity [2]. A form of exercise that may be a useful treatment for chronic conditions strongly associated with physical inactivity is high-intensity functional training (HIFT). Also referred to as extreme conditioning programs [5,6] or multimodal training [7], HIFT is a form of exercise in which a variety of coordinated multi-joint movements (e.g., weightlifting, powerlifting, gymnastics, plyometrics) are performed with varying numbers of sets, repetitions, loads, and durations. High-intensity functional training workouts are typically performed in a circuit training format at high relative intensities, with the goal of simultaneously improving all physical attributes. For a more extensive review of HIFT, readers are referred to Feito et al. [8].

High-intensity functional training has been rapidly increasing in popularity over the past decade. CrossFit^®^, a popular HIFT program, currently has over 13,000 registered gyms worldwide [9]. Based on interview data, this success may be partially associated with the physically intense challenges and development of camaraderie inherent with HIFT exercise [10,11]. These factors may allow for HIFT to be more appealing than more traditional exercise programs that include a combination of aerobic and resistance exercise [12]. Despite its rapid growth, there remains a lack of evidence to support the positive physiological adaptations to HIFT exercise that are often reported anecdotally. 

As more individuals participate in HIFT as their primary exercise regimen, more research is necessary to characterize the outcomes of this program. In addition, it is unknown if HIFT can improve fitness parameters in physically inactive adults. The purpose of this study was to characterize the physiological and fitness adaptations following eight weeks of HIFT in physically inactive adults with no prior experience to a HIFT program.

## 2. Materials and Methods

### 2.1. Participants

Twenty participants, ages 19 to 39 years, with no prior HIFT experience, and who had not performed structured physical activity more than 2 h per week for the past 12 months, were recruited. All participants completed a physical activity readiness questionnaire (PAR-Q) and medical history questionnaire to ensure there was no condition that precluded them from exercising safely. If any health conditions were noted, participants were required to obtain physician clearance prior to participation in the study. Each participant provided written informed consent and the study was approved by the university’s institutional review board. Of the 24 total workout sessions scheduled over the 8-week intervention, all participants were instructed to attend at least 22 sessions to maintain compliance. 

### 2.2. Preliminary Procedures

All testing procedures were performed on the same day, and participants could rest as needed between tests. The participants were asked to wear comfortable clothing, provide a log of dietary habits and sleep, and to refrain from strenuous physical activity, caffeine, and alcohol consumption during the 24 h preceding testing. The participants were also asked to avoid heavy meals for the 3 h preceding testing. All testing procedures were supervised by a researcher certified through the National Strength and Conditioning Association (NSCA).

Anthropometric measurements were assessed first. Body weight was measured using a digital scale (Tanita BWB-800, Arlington Heights, IL, USA), and height was measured using a stadiometer (Perspective Enterprises Model PE-AIM-101, Kalamazoo, MI, USA). Resting heart rate (HR) was measured with a chest-worn monitor (Polar Electro T31, Lake Success, NY, USA), and resting blood pressure (BP) was measured with a sphygmomanometer and stethoscope after 5 min of sitting quietly in a chair. Two BP measurements were taken 1 min apart, and the average of the two was recorded. Lean body mass and fat mass were measured by a trained technician using dual-energy X-ray absorptiometry (Lunar DXA-Prodigy, GE-Lunar, Madison, WI, USA).

Following a 5-min warm-up of light (<40% heart rate reserve [HRR]) aerobic exercise on a motorized treadmill (Star Trac E TRx, Vancouver, WA, USA) and light stretching, upper and lower body muscular strength were measured using a five-repetition maximum (5RM) test on the barbell bench press and leg press exercise (Cybex Squat Press, Medway, MA, USA), respectively. Procedures for the 5RM tests were adapted from the NSCA [13]. A 5RM protocol was selected, as it may be recommended for untrained participants [13] and is a strong predictor of 1RM with leg press (R^2^ = 0.974) and chest press (R^2^ = 0.993) exercises [14]. Upper body muscular endurance was then measured using the YMCA Bench Press Test. For this test, participants were required to perform the barbell bench press exercise at a rate of 30 reps/min to volitional exhaustion. Males lifted 36.4 kg and females lifted 15.9 kg of weight throughout the test. The test ended once the participant could no longer maintain proper cadence. Abdominal muscular endurance was measured with the One-Minute Sit-Up Test. Participants were asked to perform as many sit-ups as possible in 1 min on a standard yoga mat. Trunk and hamstring flexibility were also measured using a Sit-and-Reach Test with a flexometer (Figure Finder Flex-Tester, Novel Products, Inc., Rockton, IL, USA). For this test, participants were asked to remove their shoes and sit with their feet flat against the box. Keeping their legs extended and their hands overlapped, the participants reached forward slowly and pushed the pin on the box as far forward as possible. Participants held the terminal position for 2 s and kept the knees extended throughout.

Participants then completed a graded exercise test to volitional exhaustion. Maximal oxygen consumption (VO_2max_), a measure of cardiorespiratory fitness, was assessed using the Ball State University/Bruce Ramp protocol [15] on a motorized treadmill (Cardiac Science TM65, Waukesha, WI, USA). Participants were fitted with a HR monitor and respiratory gasses were collected using an integrated metabolic system (ParvoMedics, Sandy, UT, USA). Obtainment of VO_2max_ was determined by a plateau in VO_2_ with an increase in workload, or achieving a respiratory exchange ratio greater than 1.1 and a maximal HR within 10 beats/min of age-predicted max (220 − age).

### 2.3. High-Intensity Functional Training Intervention

The HIFT intervention was performed 3 days per week for 8 weeks at a CrossFit^®^ gym in Lewisville, TX, USA. The participants attended classes every Monday, Tuesday, and Thursday, and all received similar workout plans. The participants had a choice between morning or evening class times and were asked to consistently attend classes at the same time of day. In the event a participant missed a day, a class could be attended on a Saturday morning. Participants were contacted weekly in-person or via email regarding the progress of the exercise program. Participants were asked to report symptoms of delayed onset muscle soreness (DOMS), pain not associated with DOMS, injuries, enjoyment of the exercise program, and any other related issues. The first week of the program included three introductory classes in which the participants were taught how to properly perform all of the exercises. The gym employed this program to help ensure the safety of new clients, and to familiarize the participants with the class format. 

The classes lasted 60 min and included a dynamic warm-up, resistance exercise, a metabolic conditioning phase, and occasionally a cool-down of static stretching. The metabolic conditioning phase included aerobic and resistance exercise that was typically performed in a circuit, in which an individual attempted to complete as many rounds of the circuit as possible in a set amount of time, or attempted to complete the circuit as fast as possible. The trainer of each class utilized their best judgment to determine the proper exercise and load for each client. This ensured that clients of different skill and fitness levels could complete the workout. For example, if the participant could not perform a pull-up, the ring row exercise would be used as a substitution. While these modifications can affect each participant’s training load, it was allowed in order to maintain ecological validity. The investigators were blinded to the workouts at the gym, and the individual responsible for programming the workouts was unaware of the fitness variables that were measured in this study. This ensured that the measured fitness variables were not specifically targeted by the program. A complete list of the workouts performed during the HIFT program, organized by day and week, is found in Table A1 in Appendix A. The participants were asked to maintain their typical dietary, sleep, and physical activity habits throughout the intervention. Participants were also asked not to consume any new dietary supplements during the intervention.

### 2.4. Post-Testing Procedures

Following the 8-week intervention, post-testing procedures were completed in a similar manner as the preliminary procedures at the same time of day and by the same researcher. The participants were provided their dietary, sleep, and activity logs that were completed during the preliminary testing so these conditions could be matched. All procedures were completed 2 to 7 days following the end of the intervention.

### 2.5. Statistical Analysis

Differences between pre and post measurements were compared with dependent *t*-tests using appropriate software (IBM SPSS Statistics v.24, Armonk, NY, USA). All variables were checked for normality with the Shapiro-Wilk test, and box plots were used to check for outliers. Diastolic BP and sit-and-reach data were not normally distributed, so the Wilcoxin signed-rank test was used to analyze these variables. Statistical significance was set at 0.05. Cohen’s d effect sizes were reported and calculated as: d = (pretest score − posttest score)/pooled standard deviation.

## 3. Results

### 3.1. Participant Characteristics and Intervention Adherence 

Twenty participants were initially recruited. Two participants dropped before the study began due to the time commitment required. An additional four participants dropped due to missing more than the required number of classes, either because of illness (*n* = 2) or other commitments (*n* = 2). Therefore, 14 participants (10 women and 4 men) completed all procedures. Characteristics of these participants can be found in Table 1. Participants who completed the procedures attended an average of 23 out of the 24 scheduled workout sessions.

### 3.2. Effects on Physiological and Fitness Measures

The differences observed with all cardiorespiratory variables, body composition measures, and muscular fitness variables before and after the intervention can be found in Table 2, Table 3 and Table 4, respectively. Resting HR (*p* = 0.006) and resting diastolic BP (*p* = 0.013) were improved following the intervention, but resting systolic BP remained unchanged (*p* = 0.133). Absolute (*p* = 0.003) and relative (*p* = 0.003) VO_2max_ also increased following the intervention. Body mass did not change significantly following the intervention (*p* = 0.603) but there was a statistically significant improvement in body fat percentage (*p* = 0.023). This improvement may be explained by a statistically significant increase in lean body mass (*p* = 0.006) without a statistically significant change in fat mass (*p* = 0.227). Improvements were observed in all measures of muscular fitness. Weight lifted during the leg press 5RM (*p* < 0.001) and bench press 5RM (*p* < 0.001), repetitions completed during the YMCA Bench Press Test (*p* < 0.001) and One-Minute Sit-Up Test (*p* < 0.001), and performance on the Sit-and-Reach Test (*p* = 0.002) all improved. 

### 3.3. Qualitative Data

Anecdotally, all participants reported symptoms of DOMS throughout the intervention, with symptoms generally improving as the intervention progressed. Participants reported pain not associated with DOMS in the elbows (*n* = 1), lower back (*n* = 4), shoulders (*n* = 2), knees (*n* = 3), wrist (*n* = 1), and right quadricep (*n* = 1). Participants suggested squats, snatches, deadlifts, and burpees as possible causes of the pain. All reports of pain in these joints subsided within one week. One participant complained of sporadic, minor neck pain throughout the intervention, possibly due to a failed handstand attempt. These reports did not require any missed time from the intervention or a visit to a health professional. No significant injuries were reported. All participants in this study expressed enjoyment in the program, specifically with the social interaction, competitive atmosphere, and the physical challenges that were presented.

## 4. Discussion

The purpose of this study was to determine the physiological and fitness adaptations following eight weeks of HIFT in physically inactive adults. The major findings from this study include statistically significant improvements in cardiorespiratory fitness, body composition, muscular strength, muscular endurance, and muscular flexibility in physically inactive adults after an 8-week HIFT intervention. The results of this study may be applicable to the physically inactive young adults common in western industrialized society, and may be of practical significance for practitioners and healthcare professionals who wish to educate clients on the benefits of HIFT exercise. Readers may consult Table A2 in Appendix B for a comparison of the present study with other prominent HIFT literature.

Several variables were assessed in this study to quantify the effects of an 8-week HIFT program on cardiorespiratory fitness and additional cardiovascular stress. With regard to aerobic fitness, there is little agreement in previous research regarding the effects of HIFT. Nieuwoudt et al. [16] reported a 15.6% improvement in absolute VO_2max_ upon completing a 6-week HIFT program, although the participants were deconditioned at baseline. Goins [17] similarly reported an 11% increase in VO_2max_ in recreationally active individuals after 6 weeks of HIFT. Sobrero et al. [18] reported no change in VO_2max_ in recreationally active women after a HIFT program of similar length. Crawford et al. [19] also reported no statistically significant improvements in VO_2max_ after a 9-week HIFT program in untrained adults; however, VO_2max_ was not assessed with a metabolic cart. The participants in this study experienced a modest improvement in absolute and relative VO_2max_ of 6.3 and 5.5%, respectively. The findings in this study are similar to those observed in studies that include circuit weight training (CWT), where VO_2max_ is reported to improve approximately 5% with little to no aerobic exercise performed at a moderate- to high-intensity (e.g., running) [20]. Differences in the implementation of a HIFT program could explain these results. Goins [17] performed more running protocols and had participants exercise 5 days/week. As improvements in VO_2max_ can be activity-specific [21], a change in this measure of cardiorespiratory fitness can be attributed to the frequency of the testing modality during an exercise intervention.

An 8.5% decrease in resting diastolic BP, with no change in resting systolic BP, was observed following the intervention in this study. Two participants were not included in the analysis of blood pressure. One participant was taking antihypertensive medication before the study began and one participant’s blood pressure responses could not be obtained due to equipment failure. A 6.85% decrease in resting HR was also observed in this study. Goins [17] reported a 14% decrease in diastolic BP, with no change in systolic BP and no changes in resting HR, following 6 weeks of HIFT. Specifically, resting diastolic BP decreased by 6 mmHg in this study and by 9.3 mmHg in the study by Goins [17], which are greater reductions than those reported in many combined aerobic and resistance training studies [22]. In a recent meta-analysis, Cornelissen and Smart [22] reported that combined training programs typically result in reductions in resting diastolic BP but not resting systolic BP. These findings were confirmed in this study. This phenomenon has also been observed with CWT [23]. Further research with HIFT programs may help provide more insight into these findings.

In this study, a 2.4% decrease in body fat percentage was observed, primarily due to a 2.2% increase in lean body mass. Sobrero et al. [18] observed a 12.54% decrease in body fat percentage following 6 weeks of HIFT, which was also due to an increase in lean body mass. These findings are similar to those of traditional resistance training programs, where improvements in body fat percentage are largely attributed to increases in lean body mass with little change in fat mass [24,25]. However, Nieuwoudt et al. [16] observed a 2.5% decrease in body fat percentage following a 6-week HIFT program, primarily due to fat loss accompanied by no change in lean body mass. Feito et al. [26] reported a 4.7% decrease in body fat percentage after 16 weeks of HIFT in recreationally active adults, with a trend towards reduced fat mass. No changes in body fat percentage or body mass have also been reported in other studies that include HIFT [12,17]. Differences between body composition assessment methods, HIFT programs, physical activity history of the participants, initial body composition, and lack of dietary control could account for these differences.

The results of the present study are similar to those reported in classic studies that include circuit weight training. Circuit weight training (CWT) involves cycling through 12 to 15 repetitions of various resistance exercises with minimal rest periods between exercises (15 to 30 s). This style of training is similar to many HIFT workouts. Body fat reductions of 0.8% to 2.9% and lean body mass gains of 1.0 kg to 3.2 kg with no changes in total body mass have been reported following CWT programs [20]. High-intensity functional training may elicit similar changes in body composition versus CWT. 

Several muscular fitness variables were also assessed in this study. Goins [17] observed a 12%, 13%, and 8% improvement in the weight lifted during a 1RM deadlift, back squat, and shoulder press, respectively, after six weeks of HIFT in recreationally active individuals. Sobrero et al. [18] also observed a 6.0% improvement in the weight lifted during a 1RM bench press after a 6-week HIFT protocol in recreationally active women. Crawford et al. [19] observed a 3.6% improvement in weight lifted during the shoulder press and a 9.8% improvement in weight lifted during the back squat after nine weeks of HIFT in untrained men and women. Feito et al. [26] reported a 14.4% improvement in weight lifted during a 5RM front squat after 16 weeks of HIFT in recreationally active men and women. In the present study, a 5RM protocol was used instead of a 1RM protocol, with an 18.6% and 22.7% observed improvement in the weight lifted during the bench press and leg press exercises, respectively. Although these values are larger than those reported in previous studies, the participants in this study were not experienced with resistance training. 

Upper body muscular endurance, as assessed by the number of repetitions completed during the YMCA Bench Press Test, improved by approximately 42.3% in this study. These results are similar to Sobrero et al. [18], who reported a 45% improvement in the number of repetitions during a push-up test. A 7.5% improvement in the repetitions during the YMCA Bench Press Test following a 10-week HIFT program has been recorded [27]. Differences in study methods, population, and study compliance likely contributed to the observed differences in improvement between this study and Barfield et al. [27]. 

In recreationally active females, Sobrero et al. [18] reported no significant differences in Sit-and-Reach Test performance after a HIFT program. A 5.9% increase in reach during the Sit-and-Reach Test was observed in this study, which reached statistical significance. The differences observed in this study versus Sobrero et al. [18] were likely due to the physical activity status of the participants. 

Bench press and leg press strength are commonly tested measures in studies that include CWT. Improvements in the amount of weight lifted during a 1RM bench press and leg press range from 12.5% to 26.4% and 15.8% to 53%, respectively, following multi-week CWT programs [23,28,29,30]. The muscular strength adaptations observed during HIFT are similar to those observed during CWT. However, the participants in this study never performed the leg press or bench press exercise during the intervention. While time was spent with similar exercises (e.g., back squat, floor press), more time was spent with the Clean and Jerk and Snatch exercises and plyometrics (e.g., box jumps, jumping rope). Performing these exercises may elicit greater improvements in muscular power when compared to other training programs that include traditional resistance exercises [31]. Future research should include measures of muscular power in addition to traditional strength measures following HIFT. 

The safety of HIFT programs has been a topic of discussion [5]. Concerns have been voiced due to anecdotal evidence or case reports [32,33]. Some participants in the present study reported pain not associated with DOMS in the elbows, lower back, shoulders, knees, wrist, right quadricep, and neck. The shoulder, knee, and lower back are the most commonly reported injury sites with HIFT [34,35,36,37]; however, reports of pain in the present study were primarily acute. Reports of injuries with HIFT range from 2.1 to 3.1/1000 training hours [32,33,34,38,39]. The injury risk of HIFT may be similar to that of weightlifting and powerlifting [40], but lower than that of contact sports [41] and running [42]. Although injury incidence was not an outcome variable in this study, the authors do not find cause to discourage HIFT exercise in physically inactive adults when proper precautions are observed (i.e., adequate warm-up, proper instruction of technique, proper prescription of exercise load).

A primary limitation in this study was the lack of a control or comparison group. This study was also limited by its sample size. While the sample was quite diverse, with representation from multiple ethnicities (i.e., Caucasian, African American, Hispanic), age groups (i.e., 19 to 39 years), athletic backgrounds (most of the participants had never exercised regularly), and body fat percentages (i.e., 20.9 to 54.8%), the study was not sufficiently powered to determine how the results differed between these groups. While the study was not powered to determine differences between sexes, results were similar when females were analyzed separately. 

A general limitation in HIFT research is the inherent variability between HIFT programs. Although similar exercises and training principles are taught in facilities where HIFT exercise is performed, most coaches design their own workouts. While results from a single 8-week block at one gym are presented in this study, the results may have been dissimilar if performed at a different gym. The environment at the gym could also have affected physiological responses and adaptations to HIFT. The intervention was performed in a warehouse ventilated by portable drum fans in Texas during the summer. Exercising during the hotter times of day may elicit changes in the cardiovascular and thermoregulatory systems that are not observed in a cooler environment [43]. Finally, while most participants attended the same class time each day, some participants switched class times. Anaerobic exercise performance varies throughout the day based on circadian rhythm [44]. It is unclear if adaptations were affected by changing exercise times.

## 5. Conclusions

In conclusion, HIFT may be useful in improving health-related physical fitness parameters in physically inactive adults. Participants may experience improvements in resting HR, diastolic BP, muscular strength, muscular endurance, and lean body mass, with modest improvements in aerobic fitness and muscular flexibility. High-intensity functional training performed three days/week without dietary modification does not appear to significantly decrease fat mass in physically inactive adults. If a participant’s goal is fat loss, additional exercise and changes to dietary intake might be required [2]. Results may be similar to those following CWT programs; however, HIFT may lead to improvements in physical work capacity that are not predicted by traditional measures of physical fitness [19]. The high-intensity nature of this exercise program may result in temporary muscle soreness and acute joint pain, particularly in those new to HIFT. However, based on findings in the literature, the risk of sustaining a significant injury is low.

## Figures and Tables

**Table 1:** Characteristics of the participants before the high intensity functional training intervention.

Variable	Female (*n* = 10)	Male (*n* = 4)
Age (years)	26 ± 6	30 ± 8
Body Mass (kg)	70.71 ± 18.28	114.6 ± 43.56
Height (cm)	163.21 ± 7.53	176.03 ± 7.57
BMI (kg/m^2^)	26.49 ± 6.16	36.38 ± 11.37

Note. All values are presented as means ± standard deviation; Body Mass Index (BMI) = body mass (kg)/height (m)^2^.

**Table 2:** Cardiorespiratory measurements before and after 8 weeks of high-intensity functional training.

Variable	Pre-HIFT	Post-HIFT	*p*-Value	Δ	Effect Size d
HR_rest_ (bpm)	73 ± 12	68 ± 11 *	0.006	−6.8%	0.40
SBP_rest_ (mmHg)	112 ± 13	108 ± 12	0.133	−3.6%	0.31
DBP_rest_ (mmHg)	71 ± 7	65 ± 6 *	0.013	−8.5%	0.87
VO_2max_ (L/min)	2.53 ± 0.68	2.69 ± 0.66 *	0.003	+6.3%	−0.23
VO_2max_ (mL/kg/min)	32.51 ± 8.84	34.31 ± 8.63 *	0.003	+5.5%	−0.21

Note. All values are presented as means ± standard deviation; HR = heart rate; SB*P* = systolic blood pressure; DB*P* = diastolic blood pressure; VO_2max_ = maximum oxygen consumption; Δ = ([posttest score − pretest score]/pretest score) × 100; d = (pretest score − posttest score)/pooled standard deviation; * = significantly different from pretest score (*p* < 0.05).

**Table 3:** Body composition measurements before and after 8 weeks of high-intensity functional training.

Variable	Pre-HIFT	Post-HIFT	*p*-Value	Δ	Effect Size d
Body Mass (kg)	83.25 ± 33.02	83.52 ± 33.11	0.603	+0.3%	−0.01
Lean Body Mass (kg)	48.20 ± 13.37	49.26 ± 13.81 *	0.006	+2.2%	−0.08
Fat Mass (kg)	32.53 ± 20.45	31.92 ± 20.24	0.227	−1.9%	0.03
Body Fat (%)	37.72 ± 10.45	36.82 ± 9.84 *	0.023	−2.4%	0.09

Note. All values are presented as means ± standard deviation; Δ = ([posttest score − pretest score]/pretest score) × 100; d = (pretest score − posttest score)/pooled standard deviation; * = significantly different from pretest score (*p* < 0.05).

**Table 4:** Muscular fitness measurements before and after 8 weeks of high-intensity functional training.

Variable	Pre-HIFT	Post-HIFT	*p*-Value	Δ	Effect Size d
Leg Press 5RM (kg)	164.61 ± 54.35	201.62 ± 67.50 *	<0.001	+22.7%	−0.60
Bench Press 5RM (kg)	39.12 ± 20.15	46.43 ± 21.18 *	<0.001	+18.6%	−0.35
YMCA Test (reps)	26 ± 13	37 ± 16 *	<0.001	+42.3%	−0.74
Sit-Up Test (reps)	25 ± 9	32 ± 10 *	<0.001	+28.0%	−0.81
Sit-and-Reach (cm)	30.36 ± 11.36	32.14 ± 9.66 *	0.002	+5.9%	−0.17

Note. All values are presented as means ± standard deviation; 5RM = five repetition maximum; Δ = ([posttest score − pretest score]/pretest score) × 100; d = (pretest score − posttest score)/pooled standard deviation; * = significantly greater than pretest score (*p* < 0.05).

## Appendix A

**Table A1:** Complete list of workouts for 8 weeks of high-intensity functional training.

Week	Day 1	Day 2	Day 3
1	*Intro Class—Day 1* A. Squat B. Pushup C. Burpee D. Pull-ups E. Toes to Bar F. Shoulder Press/Push Press/Push Jerk G. Deadlift H. AMRAP 5:00: 5 Pullups, 10 Pushups, 15 Squats	*Intro Class—Day 2* A. Foam Rolling B. Back Extensions C. GHD Situps D. Dips E. Back/Front Overhead Squat F. Thruster G. Kettlebell Swings H. Tabata Kettlebell Swings, Burpees	*Intro Class—Day 3* A. Rowing B. Double Unders C. Wall Ball D. Handstands/ Handstand Pushups E. Clean F. Snatch G. 3 Rounds for time: 300 m Row, 10 Hang Power Cleans, 15 Box Jumps
2	A. EMOTM × 5: 4 Power Cleans at 75% 1RM B. Push Press: 8 at 50% 1RM, 7 at 60% 1RM, 6 at 70% 1RM, 2 × 5 at 75% 1RM C. 15-12-9 reps for time: Pull-ups, Heavy KB Swings (70# males/53# females), Burpees	A. Back Squats: 10 at 50% 1RM, 8 at 60% 1RM, 2 × 6 at 70% 1RM, 2 × 6 at 75% 1RM B. 4 Rounds for time: 400m Run, 35 HR Push-ups, 25 Box Jumps (24 in males/20 in females)	A. EMOTM × 8: Clean Pull + Clean + Front Squat 70–80% 1RM B. Hollow Holds: 5 × 30 OR Hollow Rocks 5 × 20 C. AMRAP 8:00: 16 Weighted Lunges (53# males/35# females), 8 Pull-ups
3	A. Push Press: 8 at 50% 1RM, 7 at 60% 1RM, 6 at 70% 1RM, 2 × 6 at 75% 1RM, 2 × 4 at 80% 1RM B. For Time: 20 Thrusters (95# males/65# females), 100m Suitcase Carry (53#males/35# females), 15 Thrusters, 150m Suitcase Carry, 10 Thrusters, 200m Suitcase Carry	A1. Weighted Step-ups: 5 × 6 each leg A2. Hollow to Arch: 5 × 10 − 12 on bar or rings B. AMRAP 7:00: 6 Deadlifts (225# males/155# females or 65% 1RM), 40 Double Unders	A. Snatch: 5 at 60% 1RM, 5 at 65% 1RM, 4 at 70% 1RM, 2 × 4 at 75% 1RM, 3 at 80% 1RM B. 5 Rounds for time: 250m Row, 16 Push-ups, 8 Toes To Bar, Rest:60 between rounds
4	A. Back Squats: 10 at 50% 1RM, 8 at 60% 1RM, 8 at 70% 1RM, 2 × 8 at 75% 1RM, 5 at 80% 1RM B. 3-6-9-12-15-18-21 reps for time: Pull-ups, Push Jerks (115# males/75# females)	A. Snatch: 5 at 65% 1RM, 4 at 70% 1RM, 2 × 4 at 75% 1RM, 2 × 3 at 80% 1RM B. Pendlay Row: 4 × 10 C. AMRAP 13:00: 200 m Run, 20 Burpees, 20 Thrusters (75/55)	A1. Push Press: 7 at 60% 1RM, 6 at 70% 1RM, 5 at 75% 1RM, 2 × 5 at 80% 1RM, 4 at 85% 1RM A2. Ring Rows: 5 × 8 AHAP B. EMOTM x 18: Odds: 4 Power Cleans at 70% 1RM, Evens: Calorie Row (12 male/9 female)
5	A1. Clean + Front Squat: 5 × 2 + 1 (add weight each set) A2. Strict Pull-ups: 4 × 60% 1RM B. For time: 400 m Run (85% max speed), then 20-15-10 reps of: Box Jumps (24 in male/20 in female) and V-ups, 400 m Run (85%), then 20-15-10 reps of: Overhead Lunges (45# males/25# females) and Push-ups, then 400 m Run (85%)	A1. Back Squats: 8 at 70% 1RM, 8 at 75% 1RM, 3 × 5 at 80% 1RM A2. Strict HSPU: 4 × 70% max reps B. AMRAP 16:00: 7 Power Cleans (135# males/95# females), 35 Double Unders, 7 Toes to Bar, 100 m Suitcase Carry (53# males/35# females)	A1. Split Jerks: 2-2-2-2 (increasing weight), 2 × 2 A2. Kipping Pull-ups: 4 × 7 − 10 B. EMOTM × 10: 100 m Sprint
6	A. Push Press + Split Jerk: 5 × 2+2 (Start at 75% of Push Press 1RM and add each set) B. 4 Rounds for time: 15 Calorie Row, 15 Box Jump Overs (24 in male/20 in female), Rest 2:00 C. GHD Sit-ups: 4 × 15−20	A1. Floor Press: 8 at 65% 1RM, 6 at 70% 1RM, 6 at 75% 1RM, 3 × 5 at 80% 1RM A2. Pistols: 4 × 5−10 B. AMRAP 7:00: 25 Wall-balls (20# male/14# female), 5 Deadlifts (275# male/185# female)	A. EMOTM x 7: Power Snatch + Snatch B. 8 Rounds for time: 50m Sled Drag (face sled), 30 KB Swings (53# male/35# female)
7	A1. Clean and Jerk: 1-1-1-1-1 then, 2 × 1 at 90% 1RM A2. Strict Pull-ups: 3 × 80% max reps B. 5 Rounds for time: 15 Wall-balls, 10 Burpees *10 min time cap	A. 3 Rounds for time: 800 m Run, 10 Power Cleans (185# male/125# female), 10 Front Squats, 75 Double Unders, 10 Toes to Bar, 10 Chest to Bar Pull-ups *40 min time cap B. L-sit x 1:00, Side Plank x 1:00, Plank Hold x 3:00	A1. Deadlifts: 8 at 60% 1RM, 2 × 6 at 70% 1RM, 2 × 5 at 80% 1RM A2. Kipping HSPU: 5 × 70% max reps B. 75 Calorie Row for time
8	A. Floor Press: 10 at 50% 1RM, 10 at 60% 1RM, 3 × 10 at 70% 1RM B. For time: 30 Box Jumps Overs (24 in male/20 in female), 30 Walking Lunges (each leg), 30 GHD Sit-ups, 30 Calorie Row, 30 Pull-ups, 30 Burpees, 30 Wallballs (20# male/14# female), 30 Dumbbell Snatches (40# male/25# female)	A. Back Squat: 15 at 50% 1RM, 15 at 60% 1RM, 2 × 15 at 70% 1RM B. AMRAP 5:00: 21-15-9 reps of: Thrusters (95# male/65# female), Pull-ups Rest 2:00 Then AMRAP 5:00: 15 Hang Power Snatches (95# male/65# female), 30 Double-unders	A. EMOTM × 6: 2-5 Strict Pull-ups + 2x’s Kipping B. 5-10-15-20-25 reps for time of: Hang Squat Cleans (male: 155-135-115-95-65#, female: 105-95-85-65-45#), HR Push-ups (Run 400m before each round)

Note. Intro Class = Introductory classes to teach new members the basic techniques; AMRA*P* = as many rounds/reps as possible; GHD = Glute-Ham Developer; EMOTM = every minute on the minute; # = pounds; KB = kettlebell; HR = hand release; AHA*P* = As hard as possible; HSPU = Handstand pushups; Ca*p* = time cap; 1RM = one repetition maximum; loads and exercises may be substituted based on skill level.

## Appendix B

**Table A2:** Comparisons of the present study with other prominent high-intensity functional training literature.

Study	Participants	HIFT Protocol	VO_2max_	SBP	DBP	BF%	UBMS	LBMS	UBME	LBF
Present study	Physically inactive	3 d/wk for 8 weeks	+5.5%	NS	−8.5%	−2.4%	+18.6%	+22.7%	+42.3%	+5.9%
Crawford et al. (2018)	Recreationally active and untrained	5 d/wk for 6 weeks	NS	-	-	-	+3.6%	+9.8%	-	-
Feito et al. (2018)	Recreationally active	3–5 d/wk for 16 weeks	-	-	-	−4.7%	-	+14.4%	-	-
Sobrero et al. (2017)	Physically active	3 d/wk for 6 weeks	NS	-	-	−12.5%	+6.0%	-	+45.0%	NS
Nieuwoudt et al. (2017)	Sedentary with type 2 diabetes	3 d/wk for 6 weeks	+15.6%	-	-	−2.5%	-	-	-	-
Goins (2014)	Physically active	5 d/wk for 6 weeks	+11.0%	NS	−14.0%	NS	+8.0%	+13.0%	-	-

*Note.* Males and females were recruited in all studies, with the exception of Sobrero et al. (2017), who recruited only females. HIFT = High-Intensity Functional Training; VO_2max_ = maximum oxygen consumption; SBP = Systolic Blood Pressure; DBP = Diastolic Blood Pressure; BF% = Body Fat Percentage; UBMS = Upper-Body Muscular Strength; LBMS = Lower-Body Muscular Strength; UBME = Upper-Body Muscular Endurance; LBF = Lower-Body Flexibility; NS = Not Significant; - = Not measured; all percent changes are calculated as ([posttest score – pretest score]/pretest score) × 100.
